# Buprenorphine: Far Beyond the “Ceiling”

**DOI:** 10.3390/biom11060816

**Published:** 2021-05-31

**Authors:** Rosmara Infantino, Consalvo Mattia, Pamela Locarini, Antonio Luigi Pastore, Sabatino Maione, Livio Luongo

**Affiliations:** 1 Department of Experimental Medicine, Division of Pharmacology, University of Campania “L. Vanvitelli”, 80138 Naples, Italy; rosmainfantino@gmail.com (R.I.); sabatino.maione@unicampania.it (S.M.); 2 Division of Anesthesiology, Intensive Care and Pain Medicine, ICOT Polo Pontino, Sapienza University of Rome, 04100 Rome, Italy; consalvo.mattia@uniroma1.it (C.M.); pamela.locarini@uniroma1.it (P.L.); 3 Department of Medico Surgical Sciences and Biotechnologies, Sapienza University of Rome, 04100 Rome, Italy; antonioluigi.pastore@uniroma1.it; 4 Division of Urology, ICOT Polo Pontino, Sapienza University of Rome, 04100 Rome, Italy; 5 IRCSS, Neuromed, 86077 Pozzilli, Italy

**Keywords:** chronic pain, buprenorphine, biased agonist, protean agonist

## Abstract

Chronic pain, including neuropathic pain, represents an untreated disease with important repercussions on the quality of life and huge costs on the national health system. It is well known that opioids are the most powerful analgesic drugs, but they represent the second or third line in neuropathic pain, that remain difficult to manage. Moreover, these drugs show several side effects that limit their use. In addition, opioids possess addictive properties that are associated with misuse and drug abuse. Among available opioids compounds, buprenorphine has been suggested advantageous for a series of clinical reasons, including the effectiveness in neuropathic pain. Some properties are partly explained by its unique pharmacological characteristics. However, questions on the dynamic profile remain to be answered. Pharmacokinetics optimization strategies, and additional potentialities, are still to be explored. In this paper, we attempt to conceptualize the potential undiscovered dynamic profile of buprenorphine.

## 1. Introduction

Chronic pain, including neuropathic pain, strongly affects the quality of life of patients and it remains still poorly pharmacologically managed. The molecular and cellular mechanisms highlighting neuropathic pain have not yet been clarified [1,2]. Although opioids represent the major analgesics available for the treatment of moderate and severe pain, they do not provide effective analgesia in every type of neuropathic pain [3,4]. Indeed, opioids are considered to be second- or third-choice drugs, after anticonvulsant and N-methyl-D-aspartate (NMDA) antagonists [5,6]. Due to its very high distribution in the central and peripheral nervous systems (CNS and PNS), the opioid system plays a pivotal role in modulating pain and other physiological functions and pharmacological responses, including undesired side effects. Each opioid may have a different kinetic and dynamic profile, showing varying clinical utility or risks [7]. The main problems related to chronic opioid use are respiratory depression, severe constipation, tolerance, and the risk of abuse and dependence [7,8]. Among available opioids, buprenorphine has been suggested to be advantageous for a series of clinical reasons, partly explained by its unique pharmacological characteristics.

### 1.1. The Complex Pharmacology of Buprenorphine

Buprenorphine binds mu (MOR), kappa (KOR) and delta (DOR) opioid receptors, exhibiting partial agonism towards MOR and antagonism towards KOR and DOR. With a lower affinity, it also binds opioid-like receptor 1 (OLR-1), the receptor for orphanin FQ/nociceptin [9,10,11,12]. The high-affinity binding towards MOR, with a very low dissociation kinetic, is responsible for analgesia and various adverse effects such as respiratory depression, sedation, constipation, tolerance, and addiction [13]. Although buprenorphine is traditionally classified as a partial agonist, the notion that it behaves as a full agonist in analgesia (occupying only 5–10% of MOR to obtain the analgesic effect) is nowadays widely accepted [14,15,16]. Partial agonists, which typically show a partial response compared to a full agonist, may exhibit an earlier plateau effect, referred to as a “ceiling effect”. This phenomenon is based on intrinsic activity, while it may differ depending on the involved receptors or tissues. Therefore, the enhancement of the dosage of a drug is not proportional to its increased pharmacological effect.

Most importantly, it should not erroneously be translated as “partial efficacy” [5,11]. Indeed, depending on the investigated “endpoint”, the dose–effect curve may assume different patterns. In the case of buprenorphine, whether a typical full agonist pattern is observed in analgesia, a “ceiling effect” is observed in respiratory depression [5,14,15,16]. For this characteristic, buprenorphine has drawn attention as a more manageable drug among other full opioids, although respiratory depression can occur. It has been linked to the metabolites norbuprenorphine and norbuprenorphine-3-glucuronide, which are less lipophilic than the parent molecule, and therefore less able to cross the blood–brain barrier (BBB), ensuring additional safety [5,13]. The full agonism for those receptors involved in the analgesia is not yet fully understood. The involvement of the Arylepoxamide receptors (AEAr), including a truncated 6-transmembrane protein from the MOR gene (*Oprm1*), has been suggested, suggesting a similar profile to nalbuphine and butorphanol. The deletion of this protein suppresses buprenorphine-mediated analgesia, while not morphine-mediated effect, contributing, at least in part, to incomplete cross-resistance between buprenorphine and other opioids [17,18]. However, evidence shows that other mechanisms are also implicated. It could be possible that different supraspinal signaling pathways, neither opioids nor nociceptin-mediated, contribute to analgesia. Indeed, while spinal buprenorphine-induced analgesia can be reversed by naloxone, the supraspinal analgesia persists and can be reversed by okadaic acid (Ser/Thr phosphatase inhibitor), suggesting a mechanistic difference between buprenorphine and other opioids compounds (i.e., morphine or fentanyl) [5,9].

### 1.2. Anti-Hyperalgesic Properties

Of note, another characteristic that differentiate buprenorphine from other opioids is its anti-hyperalgesic effect. This mechanism has not yet been clarified. Chronic opioid treatment leads to an upregulation of dynorphin-mediated signaling, acting on KOR and indirectly on NMDA-receptor activation, promoting pain and tolerance to analgesic effect [19]. Buprenorphine might act on this mechanism through k-receptor antagonism. In addition, the blockage of voltage-gated sodium channels (the mechanism of local anesthetics) has been postulated, replying to the tramadol and fentanyl mechanism [5,20]. Taken together, these actions might counteract the central sensitization, making buprenorphine also effective in neuropathic pain, and a candidate as a first-line treatment [5].

Taken together, these features of buprenorphine have been traced in part to specific receptor subtype selectivities or coupling to different G-proteins, which seems to be different from other MOR agonists. In addition, different tissue-specific intrinsic activities could be involved. However, many questions remain unanswered.

## 2. Beyond Partial Agonism: Biased or More?

Current receptor pharmacological theories have made possible the hypothesis of biased agonism, which makes plausible the different functioning of buprenorphine under different conditions, indicating a difference in downstream signaling.

In the classical model of G protein-coupled receptor (GPCR) activation, the binding of the agonist stabilizes the activation state of the receptor resulting in the intracellular signaling; on the contrary, antagonist binding stabilizes the inactive receptor conformation. Therefore, GPCR signal transduction is usually interpreted in a linear manner, as an “ON/OFF” dualism [21]. Nowadays, evidence shows that GPCR function is multidimensional and GPCRs can interact with multiple G proteins, β-arrestins, G protein receptor-coupled kinases (GRKs) and other effectors [22,23,24,25]. The combination of GPCR multi-dimensional transmission and the ubiquitous GPCR expression with different effect depending on tissue or CNS area involved may cause activation of undesired signaling, producing side effects.

In this context, selective activation of GPCR signaling by several ligands, able to differentially activate one pathway by sparing the others, resulted in the definition of the new concept of the biased agonist [21,24,26]. Recently, to counteract side effects as well as tolerance, biased ligands have been identified/synthesized and pharmacologically characterized for several G protein-coupled receptors (GPCRs), including MOR [27]. Nowadays, it is well established that the pharmacological stimulation of opioid receptors recruits two main signal transduction pathways: the β-arrestin2 or/and the G_0/i_-protein pathway [28,29]. β-arrestin2 recruitment induces desensitization and internalization of the receptors, while the G_0/i_-protein pathway exerts different effects, including analgesia, reduction of the gut motility, respiratory depression, depending on the opioid receptor subtype [30,31]. The first biased agonist towards MOR, as well as the better pharmacologically characterized, is oliceridine, previously named TRV130 [32,33]. This compound binds to the murine and human MOR with lower nanomolar affinities than morphine, exhibiting higher potency than morphine in the reduction of the cAMP, but lower capability for recruiting β-arrestin2 [34]. Indeed, while the oliceridine-mediated cAMP reduction was antagonized by naloxone, the oliceridine itself antagonized the recruitment of β-arrestin2 induced by the Ala2-MePhe4-Glyol5-Enkephalin (DAMGO), a full and potent agonist for Gαi and Gαo activation, as well as β-arrestin2 recruitment [26]. The biased dynamic profile, also involving G protein-related Kinases (GRKs), which, in turn, can drive the degree of bias [35,36,37,38], has been shown to also be analgesic after repeated administration without inducing tolerance, as compared to other opioids, such as fentanyl and morphine [39]. Other mechanisms involved in the biased profile include reduced respiratory depression in rodents [26,36] and humans [24,40,41,42,43], and reduced constipation [26,32,36,42].

These characteristics are very similar to buprenorphine. An important difference is that oliceridine shows a similar withdrawal syndrome when injected with the naloxone, confirming the hypothesis that the β-arrestine2 pathway is critical in tolerance but not in addiction [44,45], while buprenorphine is characterized by a lower risk of abuse and, more importantly, it can be appropriately used in detoxification protocols of opioid abuse [46,47]. A comprehensive kinetic pharmacological comparison of clinically relevant MOR agonists, including the novel biased agonist oliceridine, and buprenorphine was provided by Pedersen et al. (2020) [48]. In fact, buprenorphine showed a pharmacological biased profile in vitro, similar to that exerted by oliceridine. Based on the recent finding that binding time and kinetics of the agonist can influence the degree of bias [49], this aspect has also been investigated. Biased agonism towards beta-arrestin2 appears to be unaffected by multi-time-point kinetics in any agonists, including buprenorphine. However, buprenorphine would appear to be the only one to have a power increase in the response concentration curve on G_i_ and G_0_ protein activation, probably dependent on its binding kinetics, with an 18-fold higher receptor residence time compared to the clinical candidate biased agonist oliceridine. Moreover, as reported by Pedersen and coworkers, it seems that the selected agonists are differentially affected by G protein-coupled receptor kinase 2 and 5 (GRK2 and GRK5) expression. GRK2 and GRK5 overexpression greatly increased μ-opioid receptor internalization induced by morphine, but had only modest effects on buprenorphine- and oliceridine-induced internalization [48].

Overall, these data suggest that it cannot be ruled out that the reported beneficial clinical effects of buprenorphine, in terms of lower respiratory depression [14,50,51], are due to its low efficacy and complex pharmacology [52] rather than its biased profile [53]. Moreover, the only macroscopic difference between buprenorphine and oliceridine, aside from their “biased” profile, seems to be the dissociation kinetics on the receptor, which could potentially lead to different pharmacological effects [54].

However, it remains to be determined whether differences in therapeutic profile are caused by other parameters such as ADME properties of buprenorphine or differences in biased agonism, which seems to be independent of binding kinetics [48], suggesting a mechanism driven by receptor conformations.

The existence of ligand-directed active states of GCPRs different from, and competing with, constitutively active state receptors has been suggested. At the same time, another pharmacological entity, called “protean” agonists (after Proteus, the shape-changing Greek god), has been formulated on theoretical grounds. According to this theory, the same ligand of this class could act either as an agonist or an inverse agonist at the same GPCR, as shown with proxyfan, a high-affinity histamine H_3_-receptor ligand that exhibits this particular property [55]. The same phenomenon of a ligand-directed activated state could potentially exist in all G-protein-coupled receptors. Indeed, in addition to the H3 receptor, it has also been proposed for the CB2 receptor, D2 receptor, and α_2_-adrenergic receptors [56,57,58,59]. No hypothesis has been formulated, to the best of our knowledge, regarding the opioid receptor scenario.

In this context, identifying this phenomenon in opioid receptors could cast light on still-unexplained mechanisms and properties of opioid drugs, including buprenorphine.

## 3. Optimizing Buprenorphine Pharmacokinetics: Old Strategies and New Tricks

Buprenorphine has also shown pharmacokinetic advantages. In particular, metabolism and excretion have been shown to be very favorable in clinical practice. Although it is metabolized by CYP3A4, it is associated with less drug–drug interaction than other opioids. Moreover, it does not require dosage adjustment in the elderly, as well as being safe in patients with mild to moderate liver impairment and renal failure, even during dialysis [13,60,61,62,63].

In contrast, a major disadvantage was poor oral bioavailability (10–15%), due to the hepatic first-pass effect [64,65,66]. Sublingual formulations are available, achieving a bioavailability similar to that obtained by parenteral administration [67,68]. However, this formulation is burdened with illicit misuse, in particular snorting or injecting [61]. Buccal films have recently been formulated, and the efficacy and beneficial use of transdermal patches is well established. These formulations are made possible by the high lipophilicity of the molecule. Low-dose transdermal buprenorphine (5–10–20 μg/h) is a good step II analgesic [69]. It must be changed only once in 7 days, improving compliance. It has the advantage of fewer adverse reactions, especially gastrointestinal ones. The major side effect is reactions at the application site, such as erythema [5,13]. A major disadvantage is that transdermal patches show high variability in pharmacokinetics and take approximately 72 h to reach a steady state [70]. Slow onset of analgesia and impossibility of adjusting the initial dose may limit its utility in many cases, requiring the supplemental addition of an immediate-release full-MOR agonist [52].

It is known that the time from onset to offset of analgesia is largely dependent on distribution within the CNS [71,72,73]. Penetration through the BBB occurs rapidly, with slower migration to opioid receptor sites [74].

The recently discovered glymphatic system [75,76], a continuum between lymphatic vessels and glia in CNS, has recently been implied in several functions, including the clearance of waste molecules [75,76,77], but also mediation of the influx of exogenous molecules, distributing them to deeper areas of brain [75,78] and spinal cord [79]. In particular, a positive modulation of this system has been described to enhance brain delivery of intrathecally administered oxycodone and naloxone, with a larger distribution of the latter, more lipophilic molecule [80].

It would be interesting to know whether there is a contribution of this system to the distribution of buprenorphine in the CNS, especially with transdermal application. It would also be important to investigate whether this contribution changes among the various routes of administration, with particular attention to the transdermal one, and if modulation of the glymphatic system can modify the onset of analgesia.

## 4. Conclusions

Buprenorphine is an opioid that has a complex and exclusive pharmacology, which determine several advantages over other potent opioids. The clinical advantages of this compound, even when a full opioid agonist is co-administered (for example in breakthrough cancer pain), cannot be explained while assuming that buprenorphine is simply a partial agonist. Therefore, further pharmacological investigation is needed to better understand the real mechanism of action of buprenorphine.

## References

[1] Colloca L., Ludman T., Bouhassira D., Baron R., Dickenson A.H., Yarnitsky D., Freeman R., Truini A., Attal N., Finnerup N.B. (2017). Neuropathic pain. Nat. Rev. Dis. Prim..

[2] Finnerup N.B., Otto M., McQuay H.J., Jensen T.S., Sindrup S.H. (2005). Algorithm for neuropathic pain treatment: An evidence based proposal. Pain.

[3] Gierthmühlen J., Baron R. (2016). Neuropathic Pain. Semin. Neurol..

[4] Moisset X., Bouhassira D., Avez Couturier J., Alchaar H., Conradi S., Delmotte M.H., Lanteri-Minet M., Lefaucheur J.P., Mick G., Piano V. (2020). Pharmacological and non-pharmacological treatments for neuropathic pain: Systematic review and French recommendations. Rev. Neurol..

[5] Pergolizzi J.V., Scholten W., Smith K.J., Leighton-Scott J., Willis J.C., Henningfield J.E. (2015). The unique role of transdermal buprenorphine in the global chronic pain epidemic. Acta Anaesthesiol. Taiwanica.

[6] Smith H.S. (2012). Opioids and neuropathic pain. Pain Physician.

[7] Benyamin R., Trescot A.M., Datta S., Buenaventura R., Adlaka R., Sehgal N., Glaser S.E., Vallejo R. (2008). Opioid complications and side effects. Pain Physician.

[8] Højsted J., Sjøgren P. (2007). Addiction to opioids in chronic pain patients: A literature review. Eur. J. Pain.

[9] Ding Z., Raffa R.B. (2009). Identification of an additional supraspinal component to the analgesic mechanism of action of buprenorphine. Br. J. Pharmacol..

[10] Khroyan T.V., Wu J., Polgar W.E., Cami-Kobeci G., Fotaki N., Husbands S.M., Toll L. (2015). BU 08073 a buprenorphine analogue with partial agonist activity at μ-receptors in vitro but long-lasting opioid antagonist activity in vivo in mice. Br. J. Pharmacol..

[11] Lutfy K., Cowan A. (2004). Buprenorphine: A unique drug with complex pharmacology. Curr. Neuropharmacol..

[12] Negus S., Bidlack J., Mello N., Furness M., Rice K., Brandt M. (2002). Delta opioid antagonist effects of buprenorphine in rhesus monkeys. Behav. Pharmacol..

[13] Davis M.P. (2012). Twelve reasons for considering buprenorphine as a frontline analgesic in the management of pain. J. Support. Oncol..

[14] Dahan A., Yassen A., Romberg R., Sarton E., Teppema L., Olofsen E., Danhof M. (2006). Buprenorphine induces ceiling in respiratory depression but not in analgesia. Br. J. Anaesth..

[15] Heit H.A., Gourlay D.L. (2008). Buprenorphine: New tricks with an old molecule for pain management. Clin. J. Pain.

[16] Johnson R.E., Fudala P.J., Payne R. (2005). Buprenorphine: Considerations for pain management. J. Pain Symptom Manag..

[17] Grinnell S.G., Ansonoff M., Marrone G.F., Lu Z., Narayan A., Xu J., Rossi G., Majumdar S., Pan Y.X., Bassoni D.L. (2016). Mediation of buprenorphine analgesia by a combination of traditional and truncated mu opioid receptor splice variants. Synapse.

[18] Majumdar S., Grinnell S., Le Rouzic V., Burgman M., Polikar L., Ansonoff M., Pintar J., Pan Y.-X., Pasternak G.W. (2011). Truncated G protein-coupled mu opioid receptor MOR-1 splice variants are targets for highly potent opioid analgesics lacking side effects. Proc. Natl. Acad. Sci. USA.

[19] Vanderah T.W., Gardell L.R., Burgess S.E., Ibrahim M., Dogrul A., Zhong C.M., Zhang E.T., Malan T.P., Ossipov M.H., Lai J. (2000). Dynorphin promotes abnormal pain and spinal opioid antinociceptive tolerance. J. Neurosci..

[20] Haeseler G., Foadi N., Ahrens J., Dengler R., Hecker H., Leuwer M. (2006). Tramadol, fentanyl and sufentanil but not morphine block voltage-operated sodium channels. Pain.

[21] Costa-Neto C.M., Parreiras-e-Silva L.T., Bouvier M. (2016). A pluridimensional view of biased agonism. Mol. Pharmacol..

[22] Daaka Y., Luttrell L.M., Lefkowitz R.J. (1997). Switching of the coupling of the β 2-adrenergic receptor to different G proteins by protein kinase A. Nature.

[23] Schönegge A.-M., Gallion J., Picard L.-P., Wilkins A.D., Le Gouill C., Audet M., Stallaert W., Lohse M.J., Kimmel M., Lichtarge O. (2017). Evolutionary action and structural basis of the allosteric switch controlling β 2 AR functional selectivity. Nat. Commun..

[24] Violin J.D., Crombie A.L., Soergel D.G., Lark M.W. (2014). Biased ligands at G-protein-coupled receptors: Promise and progress. Trends Pharmacol. Sci..

[25] Zhang H., Sturchler E., Zhu J., Nieto A., Cistrone P.A., Xie J., He L., Yea K., Jones T., Turn R. (2015). Autocrine selection of a GLP-1R G-protein biased agonist with potent antidiabetic effects. Nat. Commun..

[26] DeWire S.M., Yamashita D.S., Rominger D.H., Liu G., Cowan C.L., Graczyk T.M., Chen X.-T., Pitis P.M., Gotchev D., Yuan C. (2013). AG protein-biased ligand at the μ-opioid receptor is potently analgesic with reduced gastrointestinal and respiratory dysfunction compared with morphine. J. Pharmacol. Exp. Ther..

[27] Tan L., Yan W., McCorvy J.D., Cheng J. (2018). Biased ligands of G protein-coupled receptors (GPCRs): Structure–functional selectivity relationships (SFSRs) and therapeutic potential. J. Med. Chem..

[28] Al-Hasani R., Bruchas M.R. (2011). Molecular mechanisms of opioid receptor-dependent signaling and behavior. J. Am. Soc. Anesthesiol..

[29] Mores K.L., Cassell R.J., van Rijn R.M. (2019). Arrestin recruitment and signaling by G protein-coupled receptor heteromers. Neuropharmacology.

[30] Fields H.L. (2011). The doctor’s dilemma: Opiate analgesics and chronic pain. Neuron.

[31] Raehal K.M., Schmid C.L., Groer C.E., Bohn L.M. (2011). Functional selectivity at the μ-opioid receptor: Implications for understanding opioid analgesia and tolerance. Pharmacol. Rev..

[32] Chen X.T., Pitis P., Liu G., Yuan C., Gotchev D., Cowan C.L., Rominger D.H., Koblish M., Dewire S.M., Crombie A.L. (2013). Structure-activity relationships and discovery of a G protein biased μ opioid receptor ligand, [(3-methoxythiophen-2-yl)methyl]({2-[(9R)-9-(pyridin-2-yl)-6-oxaspiro-[4.5]decan-9-yl]ethyl})amine (TRV130), for the treatment of acute severe pain. J. Med. Chem..

[33] Gan T., Wase L. (2020). Oliceridine, a G protein-selective ligand at the μ-opioid receptor, for the management of moderate to severe acute pain. Drugs Today.

[34] Azevedo Neto J., Costanzini A., De Giorgio R., Lambert D.G., Ruzza C., Calò G. (2020). Biased versus partial agonism in the search for safer opioid analgesics. Molecules.

[35] Glück L., Loktev A., Moulédous L., Mollereau C., Law P.-Y., Schulz S. (2014). Loss of morphine reward and dependence in mice lacking G protein–coupled receptor kinase 5. Biol. Psychiatry.

[36] Manglik A., Lin H., Aryal D.K., McCorvy J.D., Dengler D., Corder G., Levit A., Kling R.C., Bernat V., Hübner H. (2016). Structure-based discovery of opioid analgesics with reduced side effects. Nature.

[37] Raehal K.M., Schmid C.L., Medvedev I.O., Gainetdinov R.R., Premont R.T., Bohn L.M. (2009). Morphine-induced physiological and behavioral responses in mice lacking G protein-coupled receptor kinase 6. Drug Alcohol Depend..

[38] Thompson G.L., Lane J.R., Coudrat T., Sexton P.M., Christopoulos A., Canals M. (2016). Systematic analysis of factors influencing observations of biased agonism at the mu-opioid receptor. Biochem. Pharmacol..

[39] Mori T., Kuzumaki N., Arima T., Narita M., Tateishi R., Kondo T., Hamada Y., Kuwata H., Kawata M., Yamazaki M. (2017). Usefulness for the combination of G-protein- and β-arrestin-biased ligands of μ-opioid receptors: Prevention of antinociceptive tolerance. Mol. Pain.

[40] Singla N., Minkowitz H.S., Soergel D.G., Burt D.A., Subach R.A., Salamea M.Y., Fossler M.J., Skobieranda F. (2017). A randomized, Phase IIb study investigating oliceridine (TRV130), a novel µ-receptor G-protein pathway selective (μ-GPS) modulator, for the management of moderate to severe acute pain following abdominoplasty. J. Pain Res..

[41] Singla N.K., Skobieranda F., Soergel D.G., Salamea M., Burt D.A., Demitrack M.A., Viscusi E.R. (2019). APOLLO-2: A randomized, placebo and active-controlled phase iii study investigating oliceridine (TRV 130), a g protein–biased ligand at the μ-opioid receptor, for management of moderate to severe acute pain following abdominoplasty. Pain Pract..

[42] Soergel D.G., Subach R.A., Burnham N., Lark M.W., James I.E., Sadler B.M., Skobieranda F., Violin J.D., Webster L.R. (2014). Biased agonism of the μ-opioid receptor by TRV130 increases analgesia and reduces on-target adverse effects versus morphine: A randomized, double-blind, placebo-controlled, crossover study in healthy volunteers. Pain.

[43] Viscusi E.R., Skobieranda F., Soergel D.G., Cook E., Burt D.A., Singla N. (2019). APOLLO-1: A randomized placebo and active-controlled phase III study investigating oliceridine (TRV130), a G protein-biased ligand at the µ-opioid receptor, for management of moderate-to-severe acute pain following bunionectomy. J. Pain Res..

[44] Austin Zamarripa C., Edwards S.R., Qureshi H.N., Yi J.N., Blough B.E., Freeman K.B. (2018). The G-protein biased mu-opioid agonist, TRV130, produces reinforcing and antinociceptive effects that are comparable to oxycodone in rats. Drug Alcohol Depend..

[45] Bohn L.M., Gainetdinov R.R., Lin F.T., Lefkowitz R.J., Caron M.G. (2000). Mu-opioid receptor desensitization by beta-arrestin-2 determines morphine tolerance but not dependence. Nature.

[46] Shulman M.A.-O., Wai J.M., Nunes E.V. (2019). Buprenorphine Treatment for Opioid Use Disorder: An Overview. CNS Drugs.

[47] Wesson D.R., Smith D.E. (2010). Buprenorphine in the treatment of opiate dependence. J. Psychoact. Drugs.

[48] Pedersen M.F., Wróbel T.M., Märcher-Rørsted E., Pedersen D.S., Møller T.C., Gabriele F., Pedersen H., Matosiuk D., Foster S.R., Bouvier M. (2020). Biased agonism of clinically approved μ-opioid receptor agonists and TRV130 is not controlled by binding and signaling kinetics. Neuropharmacology.

[49] Herenbrink C.K., Sykes D.A., Donthamsetti P., Canals M., Coudrat T., Shonberg J., Scammells P.J., Capuano B., Sexton P.M., Charlton S.J. (2016). The role of kinetic context in apparent biased agonism at GPCRs. Nat. Commun..

[50] Dahan A., Yassen A., Bijl H., Romberg R., Sarton E., Teppema L., Olofsen E., Danhof M. (2005). Comparison of the respiratory effects of intravenous buprenorphine and fentanyl in humans and rats. Br. J. Anaesth..

[51] Mégarbane B., Marie N., Pirnay S., Borron S.W., Gueye P.N., Risède P., Monier C., Noble F., Baud F.J. (2006). Buprenorphine is protective against the depressive effects of norbuprenorphine on ventilation. Toxicol. Appl. Pharmacol..

[52] Pillarisetti S., Khanna I.K. (2015). Buprenorphine—An attractive opioid with underutilized potential in treatment of chronic pain. J. Pain Res..

[53] Wootten D., Christopoulos A., Marti-Solano M., Babu M.M., Sexton P.M. (2018). Mechanisms of signalling and biased agonism in G protein-coupled receptors. Nat. Rev. Mol. Cell Biol..

[54] Copeland R.A. (2016). The drug–target residence time model: A 10-year retrospective. Nat. Rev. Drug Discov..

[55] Gbahou F., Rouleau A., Morisset S., Parmentier R., Crochet S., Lin J.-S., Ligneau X., Tardivel-Lacombe J., Stark H., Schunack W. (2003). Protean agonism at histamine H3 receptors in vitro and in vivo. Proc. Natl. Acad. Sci. USA.

[56] Bolognini D., Cascio M.G., Parolaro D., Pertwee R.G. (2012). AM630 behaves as a protean ligand at the human cannabinoid CB2 receptor. Br. J. Pharmacol..

[57] Jansson C.C., Kukkonen J.P., Näsman J., Huifang G., Wurster S., Virtanen R., Savola J.M., Cockcroft V., Akerman K.E. (1998). Protean agonism at alpha2A-adrenoceptors. Mol. Pharmacol..

[58] Lane J.R., Powney B., Wise A., Rees S., Milligan G. (2007). Protean agonism at the dopamine D2 receptor: (S)-3-(3-hydroxyphenyl)-N-propylpiperidine is an agonist for activation of Go1 but an antagonist/inverse agonist for Gi1, Gi2, and Gi3. Mol. Pharmacol..

[59] Pertwee R.G. (2006). The pharmacology of cannabinoid receptors and their ligands: An overview. Int. J. Obes..

[60] Böger R.H. (2006). Renal impairment: A challenge for opioid treatment? The role of buprenorphine. Palliat. Med..

[61] Davis M.P., Pasternak G., Behm B. (2018). Treating chronic pain: An overview of clinical studies centered on the buprenorphine option. Drugs.

[62] Heel R., Brogden R.N., Speight T.M., Avery G.S. (1979). Buprenorphine: A review of its pharmacological properties and therapeutic efficacy. Drugs.

[63] Tegeder I., Lötsch J., Geisslinger G. (1999). Pharmacokinetics of opioids in liver disease. Clin. Pharmacokinet..

[64] Bullingham R.E., McQuay H.J., Moore R.A. (1983). Clinical pharmacokinetics of narcotic agonist-antagonist drugs. Clin. Pharmacokinet..

[65] Wang Y., Cipriano A., Munera C., Harris S.C. (2016). Dose-dependent flux of buprenorphine following transdermal administration in healthy subjects. J. Clin. Pharmacol..

[66] Yokell M.A., Zaller N.D., Green T.C., Rich J.D. (2011). Buprenorphine and buprenorphine/naloxone diversion, misuse, and illicit use: An international review. Curr. Drug Abus. Rev..

[67] Mendelson J., Upton R.A., Everhart E.T., Jacob P., Jones R.T. (1997). Bioavailability of sublingual buprenorphine. J. Clin. Pharmacol..

[68] Nath R.P., Upton R.A., Everhart E.T., Cheung P., Shwonek P., Jones R.T., Mendelson J.E. (1999). Buprenorphine pharmacokinetics: Relative bioavailability of sublingual tablet and liquid formulations. J. Clin. Pharmacol..

[69] Henningfield J.E., Sun W.-Z. (2015). Concluding statement—Neuropharmacological basis and clinical rationale for control of transdermal buprenorphine as a step II analgesic. Acta Anaesthesiol. Taiwanica.

[70] Kapil R.P., Cipriano A., Friedman K., Michels G., Shet M.S., Colucci S.V., Apseloff G., Kitzmiller J., Harris S.C. (2013). Once-weekly transdermal buprenorphine application results in sustained and consistent steady-state plasma levels. J. Pain Symptom Manag..

[71] Ebling W.F., Lee E.N., Stanski D.R. (1990). Understanding pharmacokinetics and pharmacodynamics through computer stimulation: I. The comparative clinical profiles of fentanyl and alfentanil. Anesthesiology.

[72] Koppert W., Ihmsen H., Körber N., Wehrfritz A., Sittl R., Schmelz M., Schüttler J. (2005). Different profiles of buprenorphine-induced analgesia and antihyperalgesia in a human pain model. Pain.

[73] Yassen A., Olofsen E., Romberg R., Sarton E., Danhof M., Dahan A. (2006). Mechanism-based pharmacokinetic-pharmacodynamic modeling of the antinociceptive effect of buprenorphine in healthy volunteers. Anesthesiology.

[74] Ohtani M., Kotaki H., Sawada Y., Iga T. (1995). Comparative analysis of buprenorphine- and norbuprenorphine-induced analgesic effects based on pharmacokinetic-pharmacodynamic modeling. J. Pharmacol. Exp. Ther..

[75] Iliff J.J., Wang M., Liao Y., Plogg B.A., Peng W., Gundersen G.A., Benveniste H., Vates G.E., Deane R., Goldman S. (2012). A paravascular pathway facilitates CSF Flow through the brain parenchyma and the clearance of interstitial solutes, including amyloid. Sci. Transl. Med..

[76] Nedergaard M. (2013). Garbage truck of the brain. Science.

[77] Iliff J.J., Chen M.J., Plog B.A., Zeppenfeld D.M., Soltero M., Yang L., Singh I., Deane R., Nedergaard M. (2014). Impairment of glymphatic pathway function promotes tau pathology after traumatic brain injury. J. Neurosci..

[78] Plog B.A., Mestre H., Olveda G.E., Sweeney A.M., Kenney H.M., Cove A., Dholakia K.Y., Tithof J., Nevins T.D., Lundgaard I. (2018). Transcranial optical imaging reveals a pathway for optimizing the delivery of immunotherapeutics to the brain. JCI Insight.

[79] Wei F., Zhang C., Xue R., Shan L., Gong S., Wang G., Tao J., Xu G., Zhang G., Wang L. (2017). The pathway of subarachnoid CSF moving into the spinal parenchyma and the role of astrocytic aquaporin-4 in this process. Life Sci..

[80] Lilius T.O., Blomqvist K., Hauglund N.L., Liu G., Stæger F.F., Bærentzen S., Du T., Ahlström F., Backman J.T., Kalso E.A. (2019). Dexmedetomidine enhances glymphatic brain delivery of intrathecally administered drugs. J. Control. Release.

